# Drosophila Ctf4 is essential for efficient DNA replication and normal cell cycle progression

**DOI:** 10.1186/1471-2199-12-13

**Published:** 2011-04-06

**Authors:** Justin A Gosnell, Tim W Christensen

**Affiliations:** 1 Department of Biology, East Carolina University, Greenville, NC 27858, USA

## Abstract

**Background:**

Proper coordination of the functions at the DNA replication fork is vital to the normal functioning of a cell. Specifically the precise coordination of helicase and polymerase activity is crucial for efficient passage though S phase. The Ctf4 protein has been shown to be a central member of the replication fork and links the replicative MCM helicase and DNA polymerase α primase. In addition, it has been implicated as a member of a complex that promotes replication fork stability, the Fork Protection Complex (FPC), and as being important for sister chromatid cohesion. As such, understanding the role of Ctf4 within the context of a multicellular organism will be integral to our understanding of its potential role in developmental and disease processes.

**Results:**

We find that Drosophila Ctf4 is a conserved protein that interacts with members of the GINS complex, Mcm2, and Polymerase α primase. Using *in vivo *RNAi knockdown of CTF4 in Drosophila we show that Ctf4 is required for viability, S phase progression, sister chromatid cohesion, endoreplication, and coping with replication stress.

**Conclusions:**

Ctf4 remains a central player in DNA replication. Our findings are consistent with what has been previously reported for CTF4 function in yeast, Xenopus extracts, and human tissue culture. We show that Ctf4 function is conserved and that Drosophila can be effectively used as a model to further probe the precise function of Ctf4 as a member of the replication fork and possible roles in development.

## Background

Reliable DNA replication during S-phase is vital for the sustainability of a cell and, ultimately, an organism. The proteins that assemble at licensed replication forks come together to form the Replisome Progression Complex (RPC). This complex must faithfully copy an organism's genome in the context of cell cycle regulation and chromatid segregation while at the same time safeguarding against DNA damage. In response to this highly integrated nature of the RPC, recent studies have generated models at the replication fork that link MCM helicase activity to primase activity and establishment of sister chromatid cohesion throughout S-phase [1,2].

The Ctf4 protein has recently become the subject of several investigations in eukaryotes due to its position as a central component of the RPC. It was initially identified in yeast during a screen for mutants affecting chromosome transmission fidelity, after which it is named [3]. The Ctf4 gene in Drosophila (CG13350) encodes a protein that is conserved across lower and higher eukaryotes. Ctf4 has been proposed to be a member of the Fork protection Complex (FPC) shown to link the MCM helicase activity to lagging-strand polymerase α-primase activity in eukaryotes [4]. While depletion studies in *Xenopus laevis *[5] have suggested its importance in the stabilization of this interaction and thus replication fidelity in general, Ctf4 has also been implicated in proper chromatid segregation and cohesion from their initial establishment in S-phase to separation in anaphase [2]. In fact, other members of the FPC have been shown to be essential for proper chromatid cohesion during S-phase, including Tim1/Tipin, which interact with Cohesin in humans [6]. This information suggests that replication fidelity is coordinated with chromatid cohesion. This study is the first to undertake phenotypic analysis of the effects of Ctf4 knockdown on development in a whole-organism, multi-cellular eukaryote. Using a GAL4/UAS-driven expression and heat-shock driven expression of RNAi targeting Ctf4, we demonstrate that CTF4 is an essential gene in Drosophila that is required for normal cell cycle progression, sister chromatid cohesion, endoreplication, and response to replication stress.

## Results

### Ctf4 is a conserved protein

**Figure 1:**
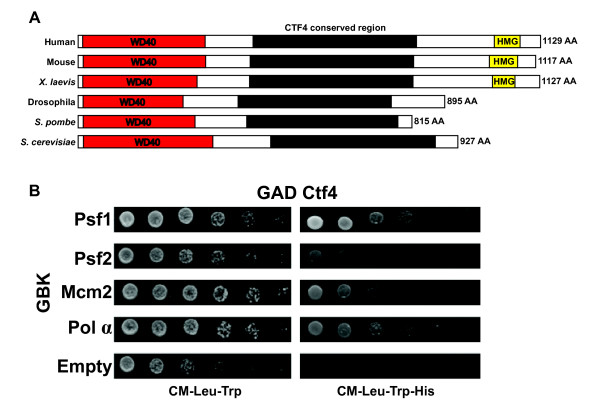
**Ctf4 is conserved across species and interacts with DNA replication proteins**. **A**. Alignment of Ctf4 from different organisms shows a conserved WD40 domain and central domain, as well as an HMG domain conserved only in vertebrates. **B**. Serial dilutions of PJ69α yeast two-hybrid reporter strain with the indicated fusion constructs. Drosophila Ctf4 fused to GAD interacts with Drosophila GBK fusions of Psf1, Psf2, Mcm2, and Pol alpha respectively as evidenced by growth on media lacking histidine, while no growth occurs in the control.

Ctf4 has been found, without exception, in a wide range of eukaryotes. The human And1/Ctf4 sequence was used to BLAST the Drosophila genome and we identified CG13350 as the Drosophila homolog of Ctf4. Multiple alignments of Drosophila Ctf4 against a range of homologs from other species reveals that protein contains a series of WD40 repeats at the N-terminus predicted to facilitate protein-protein interactions, a central conserved domain in all eukaryotes, and (in vertebrates) an HMG (High Mobility Group) box domain at the C terminus that has DNA-binding activity (figure 1A). The WD40 repeats and the central domain are conserved across all studied species. However, in vertebrates the C terminus has expanded to include an HMG box domain [7].

### Drosophila Ctf4 interacts with DNA replication proteins

Studies in yeast and Xenopus have shown a conserved role for Ctf4 in tying the activity of Polymerase α primase to the MCM helicase complex [2,5,8], and that Mcm10 recruits Ctf4 to this role [2]. In order to test whether or not Drosophila Ctf4 interacted with similar replication proteins as in other eukaryotes investigated, CTF4 was cloned from Drosophila and fused to the Gal activation domain for Yeast 2-hybrid analysis. As expected, Drosophila Ctf4 was found to interact with Polymerase α primase. In addition, consistent interaction with Psf1, Psf2 and Mcm2 was also detected (figure 1B). GAD fusion proteins tested showed no one-hybrid activity (data not shown).

### CTF4 mRNA is reduced via RNAi

**Figure 2:**
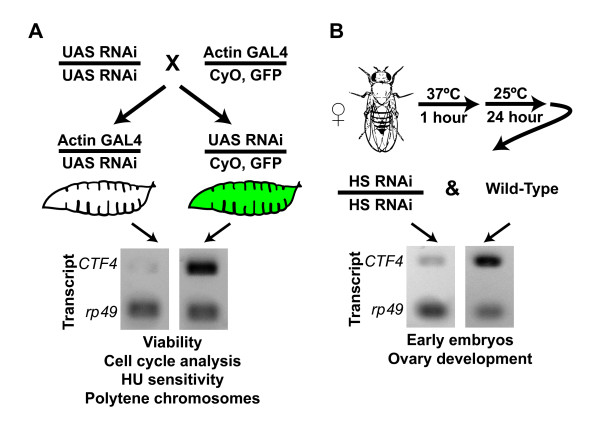
**GAL4/UAS-driven RNAi system in Drosophila for knockdown of CTF4**. **A**. Crossing scheme for the generation of CTF4 knockdown flies and rtPCR of resulting knockdown. GFP positive larvae are sibling controls for the non-glowing CTF4 knockdown larvae. Transcript levels are reduced by 87% in RNAi flies compared to sibling controls. Analysis was carried out on these groups as indicated. **B**. Alternate strategy for CTF4 knockdown using a heat-shock promoter upstream of the RNAi sequence. Analysis by rtPCR shows an 81% reduction in CTF4 transcript compared to wild-type controls.

Utilizing a Gal4 driven RNAi transgene specific to CTF4 we were able to significantly reduce levels of CTF4 transcript (figure 2A). CTF4 transcript levels were measured by rtPCR in 3^rd ^instar larvae and were found to be reduced by 87% in flies containing both the actin promoter driven Gal4 and the UAS driven CTF4 RNAi transgene as compared to the sibling control that contained only the UAS driven CTF4 RNAi transgene (figure 2A). CTF4 transcript levels were likewise reduced in females by utilizing a heat-shock promoter upstream of the CTF4 RNAi construct. CTF4 transcript levels were reduced by 81% in the females containing the RNAi construct compared to wild-type females (figure 2B).

### Ctf4 is required for viability

**Figure 3:**
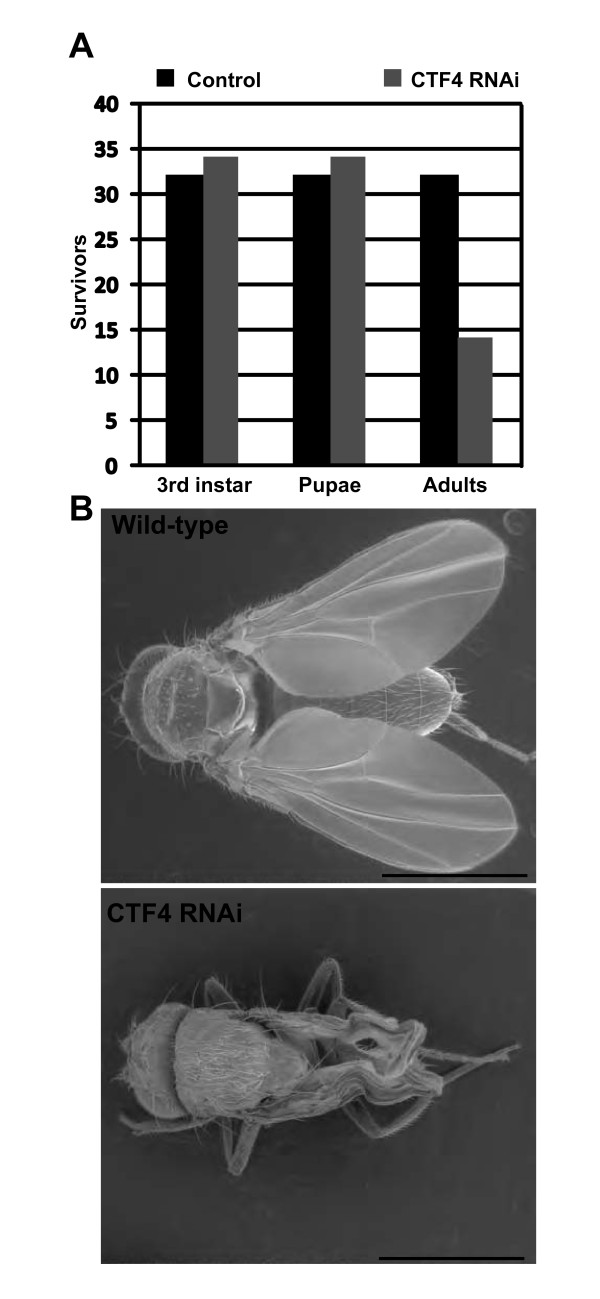
**Ctf4 is required for viability**. **A**. Graph showing survival at the third-instar, pupation, and eclosion for CTF4 knockdown and sibling control showing that CTF4 is required for successful eclosion. Maternal loading is no longer sufficient to augment knockdown of Ctf4 at pupation. **B**. SEM images of aberrant phenotype in Ctf4 knockdown eclosion survivors.

Using the Actin promoter driven Gal4 driving expression of CTF4 specific dsRNA we investigated the impact of Ctf4 depletion of the viability of Drosophila through development. We discovered that maternal loading in combination with low levels of zygotic transcription was sufficient for viability from the egg stage to pupation as the approximately 1:1 (GFP:non GFP) Mendelian ratio was observed in the progeny of UAS:CTF4 dsRNA/UAS:CTF4 dsRNA crossed to Actin:GAL4.CyO,GFP (figure 2A &3A). However, only 41.2% of Ctf4 knockdown flies succeeded in eclosing (figure 3A). Moreover, of those flies surviving this stage were all female and exhibited 100% lethality after <3 days and were severely emaciated compared to wild-type (figure 3B).

### Depletion of Ctf4 results in cell cycle progression defects

**Figure 4:**
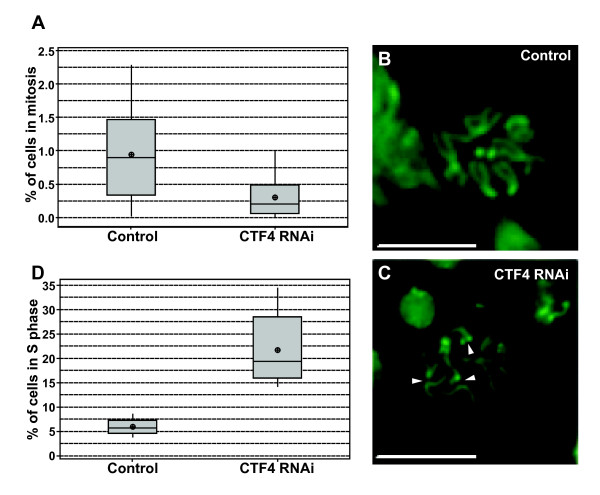
**Ctf4 is required for normal cell cycle progression**. **A**. CTF4 knockdown results in reduced mitotic index in larval brain tissue as compared to sibling control. Boxplot of mitotic indices determined from 3rd-instar wandering larva brain squashes. Larvae with the genotype RNAi-Ctf4/Actin-GAL4 (Ctf4 knockdown) yielded a significantly lower ratio of cells progressing through M-phase compared to sibling control (P = 0.004). **B-C**. Micrographs of mitotic figures from control and RNAi knockdown larval brains. Ctf4 depletion results in premature sister chromatid separation as indicated by triangles (bars represent 10 μm). **D**. CTF4 knockdown results in S phase delay. Boxplot of S-phase indices of larval brains determined by EdU incorporation showing that significantly more cells are seen in S-phase within Ctf4 knockdown larval brains compared to sibling control (P = 0.009).

Reductions in the levels of DNA replication proteins have been shown to cause cell cycle delays in Drosophila wandering 3^rd ^instar larval brain tissue [9,10]. In addition the expression level of CTF4 has been shown to be relatively high in the larval brain (Flybase ID: FBgn0033890) [11,12]. Cell cycle progression was assayed by determining the relative mitotic indices for Ctf4 depleted and control larvae. Ctf4 depletion resulted in a significant (p = 0.004) reduction in the percent of cells in mitosis (0.30% ± 0.10%) compared to the sibling control (0.94% ± 0.24%) (figure 4A). In addition to a lower mitotic index, mitotic figures were observed in the Ctf4 depleted larvae that demonstrated premature sister chromatid separation (arrows in figure 4C). No such sister chromatid separations were observed in the mitotic spreads in control larvae (figure 4B).

A reduction in the faction of cells in M phase is consistent with a possible increase in the fraction of cells in S phase in the Ctf4 depleted larvae. To directly test for a possible S-phase delay EdU (5-ethynyl-2'-deoxyuridine) incorporation and detection by fluorescence was used. EdU is a thymidine analog incorporated during DNA replication whose reactive alkyne group is detected by forming a triazole ring [13]. Quantitation of EdU incorporation in Ctf4 knockdown larval brains showed a severe S-phase delay with 21.7% ± 3.45% of cells in S phase when Ctf4 was depleted compared to 5.9% ± 0.76% in the control (p = 0.009) (figure 4D).

### Ctf4 is required for endoreplication

**Figure 5:**
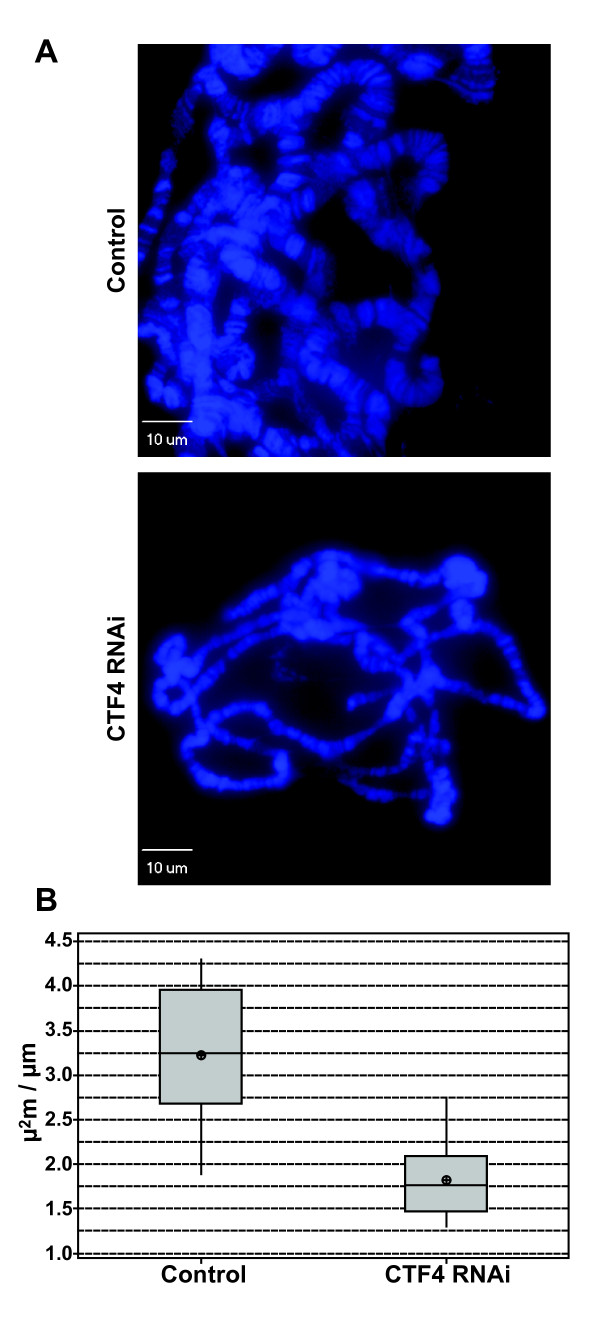
**Ctf4 is required for normal endoreplication**. **A**. Representative confocal micrographs of CTF4 knockdown polytene chromosome compared to sibling control showing thinning of chromosomes in CTF4 knockdown relative to control. **B**. Quantitation of polytene chromosomes in CTF4 knockdown compared to controls. Knockdown of CTF4 results in overall thinning of chromosome arms by an average of 43.4%.

While the larval brain tissue provides for the analysis of cells undergoing the canonical cell cycle (M-G1-S-G2), whereas the Drosophila salivary glands undergo repeated rounds of replication without mitosis producing large polytene chromosomes. Some but not all DNA replication proteins have been shown to be required for endoreplication of these tissues [9,14-16]. To investigate the impact of Ctf4 knockdown on endoreplication, Drosophila salivary glands were dissected and spread. A survey of polytene micrographs revealed a consistent thinning of polytene chromosomal arms (figure 5A), suggesting under-replication. Levels of replication were quantitated as the stained area of the chromosome/chromosome length. Overall, Ctf4 depletion resulted in polytene chromosome that were reduced in thickness by 43.4% (1.83 μm^2 ^± 0.43 μm^2^/μm) compared to the sibling controls (3.23 μm^2 ^± 0.76 μm^2^/μm) (p < 0.0000) (figure 5B).

### Ctf4 is required for early embryo cell cycles

**Figure 6:**
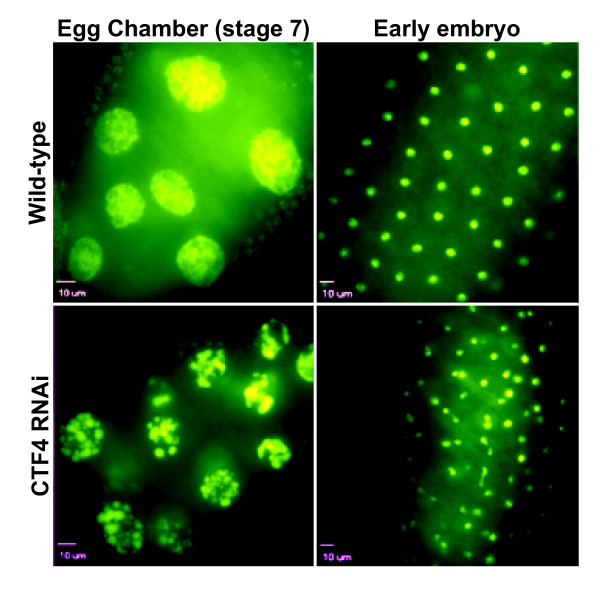
**Knockdown of CTF4 results in defects in ovary and early embryo development**. Representative confocal micrographs of egg chamber ovarioles at stage 7 in CTF4 knockdown and wild-type controls showing apoptotic-like DNA condensation in CTF4 knockdown nurse cells (right panels). Representative confocal micrographs of DNA stained with DAPI in Drosophila early embryos from both CTF4 knockdown and wild-type controls (left panels). Mitotic bridging can be seen in the Ctf4 knockdown group but was absent from controls.

Maternal loading of Ctf4 prevents the analysis of the requirement of Ctf4 in early embryo cell cycles using the Gal4 UAS:RNAi system. To circumvent maternal loading and probe the requirement for Ctf4 in early embryos we took advantage of the heat-shock promoter upstream of the CTF4 dsRNA construct and subjected transgene containing females and wild-type females to heat-shock (figure 2B) and examined the resulting embryos. Normally the syncytial nuclei divide rapidly and synchronously with no gap phase. These nuclei can become asynchronous and become linked to one another via anaphase bridges if DNA replication is compromised (Apger *et al *2010). While the wild-type control embryos appeared normal, asynchrony and anaphase bridging were observed in 69% of the embryos (cell cycles 4-10) laid by Ctf4 depleted females (figure 6).

### CTF4 knockdown results in early degeneration of egg chambers

The expression profile of Ctf4 in adult Drosophila indicates it is most actively transcribed in females within the ovaries [11,12]. These ovaries are subdivided into individual ovarioles that themselves consist of multiple egg chambers. Egg chambers are progressively more mature in the posterior direction and contain nurse cells that provide materials which nurture the oocyte and ultimately are required for the development of the embryo prior to the activation of zygotic transcription [17]. During mid-oogenisis, stages 7-8, egg chambers will naturally degenerate if the egg chamber is defective and unable to support the subsequent growth of the oocyte and embryo [18]. This pre-vitellogenic check-point is triggered in egg chambers where CTF4 has been depleted and results in degeneration of 17.4% of the stage 7-8 egg chambers (n = 4/23) as compared to no degeneration observed in the wild-type control (n = 0/31) (figure 6).

### Ctf4 is required for coping with replication stress

An important facet of the Ctf4 protein is its role in the Fork Protection Complex. This complex is thought to assist in the stabilization of paused replication forks during low nucleotide availability that occurs during replication of repetitive sequences, as well as during repair events that lead to stalling [2]. In order to assess whether or not Ctf4 plays a role in protecting paused DNA replication forks from damage we treated larvae with hydroxyurea (HU) which depletes the nucleotide pool during DNA replication leading to replication fork pausing [19,20]. Using the Gal4 UAS Ctf4 RNAi system (figure 2A) we scored surviving 3^rd ^instar larvae. Just over half (53%) of the Ctf4 depleted larvae survived relative to controls (table 1). These results indicate that normal levels of Ctf4 are required for coping with the replication stress induced by fork pausing.

**Table 1:** CTF4 knockdown larvae are sensitive to hydroxyurea

**Survival of CTF4 knockdown larvae**		
+ hydroxyurea	- hydroxyurea	Relative survival

0.56 (172)	1.06(66)	0.53

Ctf4 knockdown larvae exhibit a decrease in relative survival compared to sibling control. Survival ratios represent the percentage of glowing to non-glowing larvae. Parentheses indicate total number of larvae scored. Relative survival is the ratio of (+) hydroxyurea survival to (-) hydroxyurea survival, and shows the effect of hydroxyurea treatment on Ctf4 knockdown larvae. (Note: deviation from a 1:1 Mendelian ratio in (-) hydroxyurea group is not significant).

## Discussion

Ctf4 has been shown to stabilize helicase-polymerase association during S phase by interaction with the CMG complex. The stabilization of this association is required at the replication fork to ensure the faithful duplication of genetic information and passage through S phase [2,4,21-23]. Surprisingly, though this a conserved function across the species examined, it is not likely mediated through the highly conserved WD40 domain found in the N terminus of the protein. Instead, the C terminal portion of the protein has been implicated in interaction with the CMG complex [4]. We demonstrate that Drosophila Ctf4 interacts with Mcm2, Polymerase alpha primase, and members of the GINS complex. These interactions are consistent with a central role for Ctf4 in coordinating the helicase-polymerase association during S phase. It has been reported in budding yeast that Ctf4 interacts only with the GINS complex via interaction with Sld5 [23]. Drosophila Ctf4 appears to be different in that it interacts with the Psf1 and Psf2 subunits of the GINS complex. Given the structural similarities between the subunits of the GINS complex [24] it is not unreasonable to speculate that specific subunit association of Ctf4 may be fluid over evolutionary time.

Ctf4 has been repeatedly implicated in proper sister chromatid cohesion [3,25-28]. We show here that when Ctf4 is depleted in Drosophila, premature sister chromatid separation is observed. Our observations are made in tissues that are normally progressing through the cell cycle and are not treated with mitosis inhibitors which can cause ectopic sister chromatid separation to occur.

Research using unicellular eukaryotes, tissue culture, and Xenopus egg extracts has clearly shown a role for Ctf4 in DNA replication [4,22,26,29]. We demonstrate here, for the first time that within the context of a multicellular metazoan, Ctf4 is required for normal S-phase progression and viability through development. Moreover, we show that Ctf4 is required for endoreplication, ovary development, and early embryo cell cycles.

Previous research has indicated that there is a link between Ctf4 and Mcm10 [2,21]. Indeed both Mcm10 and Ctf4 appear to share many of the same functions in that they both coordinate aspects of the CMG complex and Polymerase alpha [2,10,21,30-37]. Using the yeast two-hybrid system we could not confirm a direct interaction between these two Drosophila proteins (data not shown). However it is interesting to note that depletion of Mcm10 by use of a hypomorphic allele results in similar S phase delays, early embryo anaphase bridges, and defects in ovary development [9]. In addition Mcm10 like Ctf4 has been shown to interact with the GINS complex in Drosophila [10]. Curiously, the knockdown of Mcm10 in Drosophila only results in a partial lethality and fly stocks may be kept homozygous for the hypomorphic MCM10 allele [9]. Within this context it is also interesting to note that conserved domain analysis of the Ctf4 consensus sequence reveals a cryptic Mcm10 like domain in the C terminal (pfam09332, E value = 3.4) [38]. The literature, combined with our observations, suggest that Mcm10 and Ctf4 may share some redundant functions with respect to coordination at the replication fork. If this is the case then the foundation laid by our work will inform double mutant analysis of these two genes leading to a better understanding of how replication fork progression complexes are coordinated and regulated.

## Conclusions

Recent investigations into Ctf4 have placed it as an integral member of the DNA replication machinery with respect to coordinating helicase and polymerase activity. Here with lay the groundwork for studying the role of this protein within the context of a multicellular metazoan. We have found that this highly conserved protein interacts with a member of the Mcm2-7 helicase, members of the GINS complex, and DNA pol alpha. Moreover, we demonstrate efficient RNAi knockdown of CTF4 in Drosophila and use these animals to show that CTF4 is required for viability, sister chromatid cohesion, S phase progression, endoreplication, ovariole development, early embryo cell cycles, and coping with replication stress. Taken altogether, CTF4 continues to be a key protein in our understanding of the underlying processes required for cell viability and organismal development.

## Methods

### Fly Husbandry/Stocks

Drosophila lines were acquired from the Bloomington Fly stock center, the Exelixis Drosophila Stock Collection at Harvard Medical School, and the Vienna Drosophila RNAi center. These strains consisted of: wt (Flybase ID: FBst0006326), w[1118]; P{GD4433}v44474 (GAL4/heat-shock inducible CTF4 RNAi, FlyBase ID: FBst0465598), P{Act-GAL4.U} (GAL4 reporter/driver, Flybase ID: FBtp0039579), P{Act-GAL4.U}/CyO, GFP derived from FBtp0039579 and FBba0000585. All stocks were kept over Drosophila K12 media (US Biological # D9600-07B) at room temperature.

### Yeast 2-hybrid

A modified Clonetech Matchmaker™ Yeast 2-hybrid system was used to detect interactions using yeast strain PJ69α as per [9] with Gateway-modified Clonetech plasmids pGBKT7 (bait) and pGADT7 (prey). Gateway entry vectors used in the construction of 2-hybrid plasmids were made from amplified Drosophila cDNA and were sequence-verified. The final destination vectors were also verified to establish that the sequence was in-frame and that no mutations were introduced in the process. Dilution series (1:5) were plated to quantify strength of interaction.

### Early Embryos

Heat-shock-inducible RNAi adult females (3-7 days post-eclosion) were incubated above yeast paste for 24 hours before the heat-shock procedure. They were then acclimated at 30°C for 30 minutes in a water bath, heat-shocked at 37°C for 1 hour and finally incubated over yeast paste a second time for 24 hours. Females were then allowed to lay eggs over grape agar with a thin film of yeast paste (1:1 baker's yeast:dH_2_O) for 8 hours. Embryos were collected and analyzed according to [9] with an Olympus IX81 Motorized Inverted Microscope with Spinning Disk Confocal controlled by the SlideBook™ software.

### Polytene Chromosomes

Third-instar wandering larvae were dissected in 1XPBS pH 7.2 with 1% PEG 8000 and salivary glands isolated and fixed with 50% acetic acid, 2-3% lactic acid, 3.7% formaldehyde. Glands were transferred to slides with siliconized cover slips and spread using periodic compression with the tip of a pencil. Spreads were then compressed with 15 Nm using a precision vise. Squashes were then frozen in liquid N_2 _and the cover slips were afterward removed. After rinsing with ethanol and subsequent drying, squashes were mounted with Vectashield™ containing DAPI and imaged using an epi-fluorescence microscope. Images were analyzed for average area of chromosome arms using quantitation software included in the Adobe Photoshop CS4 suite. Three 15.6 μm lengths per image (n = 10) were quantitated for area in square μm. A boxplot graph was generated using the average area per fixed length for each image using the Minitab™ software package.

### Adult Ovary Dissection and Analysis

Adult female Drosophila were heat-shocked using the procedure outlined above and ovaries were isolated by dissection following the second 24-hour yeast paste incubation. Ovarioles were teased apart and fixed in 4% Formaldehyde PBX (PBS + 0.1% Triton X-100) for 20 min. Ovaries were then stained for 5 min with 1 ug/mL DAPI in PBS. Ovaries were then washed 3X for 5 min in PBX, followed by a 1 hour PBX wash and finally 3 10 minute PBX washes. Ovaries were mounted using Vectashield™ and imaged using confocal optical sectioning microscopy.

### Mitotic Indices

Glowing and non-glowing third-instar larvae were separated and dissected in PBS/PEG solution. Brains were isolated and incubated at room-temperature for 10 minutes in a hypotonic solution consisting of 0.5% sodium citrate, then incubated in a 11:11:2 mixture of acetic acid:methanol:water for 20 seconds. The squashes were then prepared by placing the brains on individual slides, applying a siliconized coverslip to each, and sandwiching the coverslip under a second slide. A precision vise was then used to apply 15 Nm of force to the sandwiches for 2 minutes. The squash preparations were then frozen with liquid nitrogen, rinsed with ethanol, and dried before finally receiving a new coverslip over Vectashield™ with DAPI.

The brain squashes were analyzed by capturing 10 random fields under 600X magnification that are moderately populated (between 100 and 300 cells in view) for each of 10 slides, making for a total of 100 pictures. The Minitab™ software package was used to generate a box plot graph of the mean indices from each slide.

### EdU Incorporation

To determine the percentage of cells in S-phase for each group, the Click-It^® ^reaction kit from Invitrogen (Cat. # C10337) was used to detect EdU incorporation within larval brains [10]. These were dissected in fresh Grace's unsupplemented cell culture medium. An equal volume of 200 mM EdU solution in DMSO was added to the well and brains from each strain were incubated for 30 minutes in the dark at room temperature. Following incubation the liquid was removed from each well and the brains were rinsed three times with 1X PBS. They were then treated with a hypotonic solution of 0.5% sodium citrate for 10 minutes to expand the cells. Brains were then incubated in a 11:11:2 mixture of acetic acid:methanol:water for 30 seconds. Brains were squashed and frozen as per the above procedure, and squashes were rinsed with 3% BSA in 1X PBS. Squashes were then treated with 0.1% TritonX in PBS for 15 minutes at room temperature in the dark. The liquid was removed and the slides were rinsed twice with 1X PBS. Squashes were then incubated in the Click-It^® ^reaction cocktail as per the manufacturer's instructions for 30 minutes. The squashes were rinsed twice with the reaction rinse buffer provided by the manufacturer. After removing the rinse buffer, the counter stain Hoescht 33342 was prepared as per the manufacturer's instructions for nuclear visualization and applied for 10 minutes. The Hoescht solution was then removed and the squashes were washed four times with 1X PBS. Squashes were mounted in Vectashield™ for fluorescence and imaged using confocal microscopy. Analysis was carried out by capturing 5 random fields under 600X magnification for each of 5 slides for each group. Incidence of S-phase was detected by scoring EdU-positive nuclei. The Minitab™ software package was used to generate a box plot graph of the mean indices from each slide.

### Deoxyribonucleotide Depletion by Hydroxyurea in Ctf4 Knockdown Larvae

Hydroxyurea (HU) (MP Biomedicals, LLC) was added to Drosophila K12 media at a final concentration of 10 mM. A non-mutagenic cross vial was prepared and adult Drosophila allowed to mate and incubated for 2 days before transfer to the HU-containing vial. Eggs were laid for 2 days and parents were transferred to successive new HU-containing vials to obtain statistically reliable numbers. Third-instar wandering larvae were scored and a ratio determined between Ctf4 knockdown survivors and sibling control survivors as in [39].

## Authors' contributions

JAG carried out the experiments in this study, participated in the design and analysis of the experiments, and drafted the manuscript. TWC conceived of the study, and participated in its design and coordination and helped to draft the manuscript. All authors read and approved the final manuscript.
